# Management of ascites due to gastrointestinal malignancy

**DOI:** 10.4103/0256-4947.55167

**Published:** 2009

**Authors:** Muhammad W. Saif, Imran A. P. Siddiqui, Muhammad A. Sohail

**Affiliations:** From the Department of Medical Oncology, Yale University School of Medicine, New Haven, Connecticut, USA

## Abstract

Ascites is the pathological accumulation of fluid within the abdominal cavity. The most common cancers associated with ascites are adenocarcinomas of the ovary, breast, colon, stomach and pancreas. Symptoms include abdominal distension, nausea, vomiting, early satiety, dyspnea, lower extremity edema, weight gain and reduced mobility. There are many potential causes of ascites in cancer patients, including peritoneal carcinomatosis, malignant obstruction of draining lymphatics, portal vein thrombosis, elevated portal venous pressure from cirrhosis, congestive heart failure, constrictive pericarditis, nephrotic syndrome and peritoneal infections. Depending on the clinical presentation and expected survival, a diagnostic evaluation is usually indicated as it will impact both prognosis and the treatment approach. Key tests include serum albumin and protein and a simultaneous diagnostic paracentesis, checking ascitic fluid, WBCs, albumin, protein and cytology. Median survival after diagnosis of malignant ascites is in the range of 1 to 4 months; survival is apt to be longer for ovarian and breast cancers if systemic anti-cancer treatments are available.

The word ascites is of Greek origin (askos) and means bag or sac. Ascites is defined as the pathological accumulation of excessive fluid within the peritoneal cavity.^1^ Ascitic fluid can put pressure on the diaphragm and cause difficulty in breathing. Healthy men have little or no intraperitoneal fluid, but women may normally have as much as 20 mL depending on the phase of the menstrual cycle. Malignant ascites, the subject of this review, is a manifestation of end-stage events in a variety of cancers and is associated with significant morbidity. Malignant ascites accounts for about 10% of all cases of ascites and is usually caused by ovarian, endometrial, breast, esophageal, gastric, colorectal, lung, pancreatic, hepatobilliary and primary peritoneal carcinomas.^2^–^4^ Sometimes ascites is the sole manifestation of internal malignancies.

## Pathophysiology

**Figure 1:**
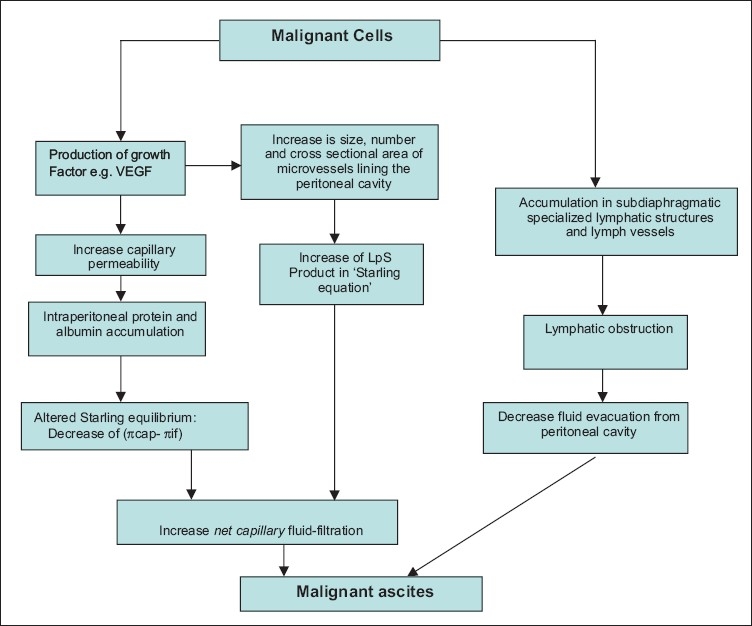
Pathophysiology of ascites.

The most common causes of ascites are related to portal hypertension, which is usually related to liver cirrhosis. Although lymphatic obstruction is considered the major pathophysiologic mechanism behind the formation of ascites, recent evidence suggests that immunomodulators, vascular permeability factors and metalloproteinase contribute significantly to the process (Figure 1).^1^ The most acceptable theory for ascites formation is peripheral arterial vasodilatation leading to underfilling of circulatory volume.

## Clinical manifestations

The usual clinical presentation is a protuberant abdomen with discomfort, difficulty in breathing, fever and pain. Sometimes the hidden GI malignancy presents with ascites only. It is known that about 50% of patients with malignant ascites present with ascites at the initial diagnosis of their cancer.^5^,^6^ The onset and progression of malignant ascites is associated with deterioration in quality of life (QoL) and a poor prognosis. According to the International Ascites Club, severity is classified as grade 1, 2 or 3 (Table 1).^7^–^9^ Based on associated complications like spontaneous bacterial pneumonitis (SBP) or hepatorenal syndrome (HRS) and therapeutic response, it can also be classified, as uncomplicated, complicated and refractory ascites.

**Table 1:** Grades of ascites.

Severity	
Grade 1 (mild)	Not clinically evident, diagnosed on ultrasound
Grade 2 (moderate)	Proportionate sensible abdominal distension
Grade 3 (severe)	Noticeable tense distension of abdomen
Uncomplicated^8^	Not infected or associated with HRS
Refractory^9^	Cannot be mobilized, early recurrence after LVP, not prevented satisfactorily with medical treatment (after 1 week)
Diuretic-resistant	No response to intensive diuretic treatment
Diuretic-intractable	Drug-induced adverse effects preclude diuretic treatment

## Diagnosis

### Lab findings

Routine blood work may be inconclusive, while some tests suggest specific etiologies. A prothrombin time (PT), activated partial thrombin time (APTT) and international normalized ratio (INR) must be done prior to paracentesis in all patients. Although serum tumors markers have low diagnostic specificity, they can be used for early detection. CEA antigen is used to detect relapses of colorectal cancer, but is also expressed in pancreatic, lung and breast cancers.^9^ Similarly, levels of cancer antigen 125 may be elevated in ovarian, pancreatic, lung or breast cancer.^9^ Ascitic fluid analysis is essential for the diagnosis of malignant ascites (Table 2).

**Table 2:** Analysis of ascitic fluid.

Routine tests	Optional tests	Unusual tests
Cell count and differential	Glucose concentration	Tuberculosis smear and culture
Albumin concentration	LDH concentration	Cytology
Total protein concentration	Gram stain	Triglyceride concentration
Culture in blood culture bottles	Amylase concentration	Bilirubin concentration

### Fluid analysis

Ascitic fluid analysis is essential for the diagnosis of malignant ascites. Exudative or transudative ascities on the basis of total protein content (≥2.5 or <2.5 g/dL, respectively,^10^ is hampered by a large overlap between malignant and non-malignant ascites. Up to 25% of patients with cirrhosis (mostly those with cardiac cirrhosis) can have high protein levels in ascites, and 18% of malignant ascites can be low in protein levels by nature.^11^

The serum-to-ascites albumin gradient (SAAG) accurately identifies the presence of portal hypertension and is more useful than the protein-based exudate/transudate concept.^12^ The SAAG is easily calculated by subtracting the ascitic fluid albumin value from the serum albumin value, which is obtained on the same day. The presence of a gradient of >1.1 g/dL (>11 g/L) indicates that the patient has portal hypertension with 97 percent accuracy.^12^ A gradient <1.1 g/dL (<11 g/L) indicates that the patient does not have portal hypertension.^12^ The SAAG need not be repeated after the initial measurement (Table 3).

**Table 3:** Classification of ascites by serum albumin ascites gradient.

High albumin gradient (SAAG >1.1)	Low albumin gradient (SAAG <1.1)
Cirrhosis	Peritoneal carcinomatosis
Alcoholic hepatitis	Peritoneal tuberculosis
Congestive heart failure	Pancreatitis
Massive hepatic metastasis	Serositis
Constrictive pericarditis and Budd-Chiari syndrome	Nephrotic syndrome

Excellent discrimination between ascites due to liver disease or malignancy is reportedly provided by ascitic fluid fibronectin (sensitivity, 100%; specificity, 100%) and cholesterol levels,^12^,^13^ although the origin of fibronectin is unclear. The gold standard for the diagnosis of malignant ascites is the presence of tumor cells in the ascetic fluid. Immunohistochemical staining combined with conventional cytologic examination increases the diagnostic sensitivity.^12^

## Management

Management of patients with ascities in GI malignancies is controversial. There are different approaches to the treatment of malignant ascites, ranging from symptomatic relief with simple drainage procedures to chemotherapy and debulking surgery aimed at treating the underlying cancer. QoL and possibly the survival of patients with malignant ascites may be improved with the increasing availability and use of appropriate and potent combination chemotherapy.^14^ The onset and progression of malignant ascites is associated with deterioration in QoL and a poor prognosis.^14^ There are, however, no generally accepted evidence-based guidelines for evaluation and treatment of this condition. There are also no clinical predictors that identify cancer patients who will develop this distressing entity; hence, there are no preventive measures for its development. Malignant ascites presents with a multitude of symptoms including abdominal distension, respiratory embarrassment and early satiety, swelling of limbs, impaired mobility, nutritional deficiencies and its effects, the management of which requires prompt yet effective relief of symptoms with an eye on reducing recurrence. A logical approach is to individualize treatment. The rationale in the management of malignant ascites involves consideration of survival and QoL issues. Palliative techniques play an important role in the reduction of symptoms, which bear a direct correlation to patient satisfaction and therapeutic choices.^14^,^15^

In a random sample of 80 physicians practicing in Canada, physicians were questioned on their use of different modalities in management of malignant ascites and preferences based on attitudes toward efficacy of various treatments.^14^ The most commonly used means of managing malignant ascites was paracentesis, which was also felt to be the most effective by the group surveyed. After paracentesis, diuretics and peritoneovenous shunting were used most frequently, but there was no apparent consensus as to their effectiveness.^14^ A survey by Lee and colleagues showed that paracentesis and diuretics were the most commonly used procedures in management of malignant ascites followed by peritoneovenous shunts, diet measures and other modalities like systemic or intraperitoneal chemotherapy.^15^ Symptom-based questionnaires have helped in evaluating the symptomatology and effectiveness of abdominal paracentesis.^15^ Commonly used ones include the Edmonton Symptom Assessment System-Ascites Modification (ESAS:AM), the Memorial Symptom Assessment Scale-Short Form, the European Organization for the Research and Treatment of Cancer (EORTC) Core Quality of Life Questionnaire (QLQ-C30), and the EORTC Core Quality of Life Questionnaire, 26-item pancreatic cancer module (QLQ-PAN26).^15^ Most patients (78%) report that their symptoms improve after paracentesis (Table 4).

**Table 4:** Improvement following paracentesis.

Symptom	Improvement score (%)
Abdominal bloating	42–54
Anorexia	20–37
Dyspnea	33–43
Insomnia	29–31
Fatigue	14–17
Mobility	25

Subscales that included the most distressing symptoms were most responsive.^15^ The amount of fluid removed (median, 3.5 L; range, 0.3 L to 9.7 L) did not correlate with symptom improvement (r=.29, *P* =−.10). All questionnaires showed strong sensitivity, validity and reliability. It has been suggested that for future clinical trials of symptomatic ascites, the QLQC30 and the ESAS:AM together, or the QLQ-C30 with the addition of the QLQ-PAN26 ascites and abdominal pain subscales could be used.^16^

### Diet

Low sodium diet is the first step towards the management of ascites. It is believed to reduce the associated water retention and help reduce edema. Long-term sodium restriction has been shown to reduce recurrences and prolong the symptom-free period.^17^

### Diuretic therapy

There are no randomized controlled trials assessing the efficacy of diuretic therapy in malignant ascites. Diuretic use in managing malignant ascites is inconsistent among physicians. A survey by Lee and colleagues showed that diuretics were used by 61% of physicians treating malignant ascites (27/44), but was felt to be effective by only 45% (20/44).^18^ Phase II data suggest that the efficacy of diuretics in malignant ascites depends on plasma renin/aldosterone concentration.^19^ In a study by Greenway and colleagues,^20^ 13 of 15 patients responded to spironolactone (doses varying from 150 to 450 mg) and plasma renin activity was raised in all of 5 patients in whom it was measured (Table 5).

**Table 5:** Diuretics: Mode of action and toxicity profile.

Diuretic class	Examples	Mechanism of action	Site of action	Side effects
Loop diuretics	Furosemide, bumetanide, torsemide, ethacrynic acid	Inhibit sodium reabsorption at the Na-Cl-2K carrier	Medullary and cortical aspects of the thick ascending limb	Hypovolemeia, Hponatremia, Hypokalemia, Hypochloremia, Hypocalcemia, Hypomagnesemia, Metabolica alkalosis, teratogenicity

Thiazide diuretics	Hydrocholorthiazide, chlorthalidone, amiloride	Inhibit NaCl reabsorption in Na-Cl cotransporter and, to a lesser degree, parallel Na-H and Cl-HCO3 exchangers	Distal tubule the connecting segment at the end of the distal tubule	Hypokalemia, Hypochloremia, Hypomagnesemia, Hypercalcemia

Potassium-sparing diuretic	Amiloride, triamterene spirinolactone, eplerenone	Inhibit sodium entry through the aldosterone sensitive sodium channels (Na-K-H+ exchange)	Principal cells in the cortical collecting tubule (and possibly in the papillary or inner medullary collecting duct	Gynaecomastia, Hyperkalemia, Endocrine abnormalities.

Carbonic anhydrase inhibitors	Acetazolamide, dorazolamide	Inhibits activity of carbonic anhydrase	Proximal tubular cells.	Metabolic acidosis, neuropathy

Osmotic diuretic	Mannitol	A non-reabsorbable polysaccharide that acts as an osmotic diuretic, inhibiting sodium and water reabsorption	Proximal tubule and more importantly, the loop of Henle	Hypovolemia, dehydration

The SAAG gradient could serve as a guideline to determine response to diuretic therapy. In the prospective study by Pockros and colleagues,^19^ a response to diuretics was seen in patients with ascites due to massive hepatic metastases who had a serum-ascites albumin gradient >1.1 g/dL (congruent to the serum-ascites albumin gradient of patients with benign ascites due to liver cirrhosis), whereas patients with ascites caused by peritoneal carcinomatosis or chylous malignant ascites who had no portal hypertension and a serum-ascites albumin gradient <1.1 g/dL did not respond to diuretics.

### Paracentesis

Available data show good, although temporary relief of symptoms related to the build-up of fluid in about 90% of patients managed by paracentesis. There is no consensus on fluid withdrawal speed. Several durations have been reported, varying from 30-90 min^21^ to 19-24 hours.^22^ Possible complications of paracentesis include secondary peritonitis, pulmonary emboli and hypotension.^23^ Fischer reported about 300 cases of abdominal paracentesis for malignant ascites where 5% dextrose was infused intravenously simultaneously and no episodes of severe hypotension were recorded.^24^ Endoscopic ultrasound-guided paracentesis (EUS-P) is highly sensitive and specific for diagnosing malignant ascites.^24^ The finding of malignant ascites significantly alters patient management, so an active search for ascites and use of EUS-P should be incorporated into the diagnosis and staging of upper GI and pancreaticobiliary tumors.^24^ The sensitivity, specificity, positive predictive value, and negative predictive value of EUS-P for diagnosing malignant ascites was 94%, 100%, 100%, and 89%, respectively.^25^ Studies in the context of liver disease showed that up to 5 L can be removed quickly without risk of significantly affecting plasma volume or renal function.^26^–^28^ Stephenson and colleagues retrospectively analyzed 30 paracenteses in 12 patients with malignant ascites after implementing a guideline allowing up to 5 L fluid to drain without clamping and giving intravenous fluids only when specifically indicated. In the analyzed 30 paracenteses, intravenous fluids or blood products were given only in 6 procedures and there was no case of symptomatic hypotension.^29^

McNamara did a prospective study in the context of malignant ascites, observing 48 paracenteses in 44 patients to evaluate how much fluid needs to be drained for symptom relief. The results suggest that a significant improvement of the symptoms of abdominal pressure occurs with the removal of few liters (range 0.8-15 L, mean 5.3 L, median 4.9 L).^30^ There are no randomized trials comparing paracentesis with the use of diuretics in the management of malignant ascites. A phase II study suggests that installation of Iscador M (visucs albumin extract) into the peritoneal cavity may reduce the need for repeated punctures.^31^ This offers significant hope for patient compliance given the nature of recurrence of ascites and the resultant frequent hospital visits.

### Peritoneovenous shunts

There are two main types of peritoneovenous shunt (PVS) systems, the Le Veen shunt^32^ and the Denver shunt.^33^ The Le Veen shunt drains ascitic fluid into the superior vena cava by a one-way valve opening at a pressure of 3 cm of water. With the Denver shunt, the valves open at a positive pressure gradient of about 1 cm of water, preventing reflux. There have been no prospective randomized studies comparing the patency rates of the two systems in malignant ascites.^34^ Souter and colleagues evaluated 43 patients with malignant ascites, 16 receiving a Denver Shunt, 27 receiving a Le Veen shunt. They observed that shunt occlusion was more common with the Denver Shunt, but the two groups of patients were not selected at random and therefore may not be comparable.^35^ LeVeen and Denver peritoneovenous shunt complications other than patency are comparable. The Le Veen is preferred for its superior patency in cirrhotic patients with intractable ascites. Hemorrhagic ascites and ascitic fluid protein content greater than 4.5 g/L are considered contraindications for shunting because of the higher risk of shunt occlusion.^35^ Loculated ascites, portal hypertension, coagulation disorders and advanced cardiac or renal failure are also contraindications.^35^ Although clinical observations and findings at necropsy indicate that peritoneovenous shunting does not result in the establishment of clinically important hematogenous metastases,^36^ some studies have shown that tumor cells infused into the central venous system can lead to massive early metastases.^37^ Reported median survival of patients with malignant ascites varies between 52 and 266 days,^34^ indicating that patient selection assumes paramount importance in deciding upon further management. In all reported studies, patients with ovarian and breast cancer who undergo peritoneovenous shunting have the best response rate (>50%) whereas the response rate in patients with gastrointestinal cancers is far worse (10% to 15%).^37^ Because of poor prognosis, it is agreed by most authors that shunt insertion is contraindicated in patients with malignant ascites due to gastrointestinal cancer.^14^ An insertion of a shunt is associated with potentially fatal side-effects and costs in terms of time and money, considering that patients need to be monitored closely for at least 24 hours after operation with a central venous pressure line to monitor fluid balance. Therefore a shunt should only be used when other treatment options like diuretics have failed and when the life expectancy of the patient is long enough to derive benefit. There is no consensus on the time span; some authors advocate more than one month,^38^ while others suggest an expected survival of more than 3 months.^39^,^40^ The use of shunts has to be balanced by the potential risks of this procedure.

### Tenckhoff catheter

This catheter is surgically placed through the wall of the abdomen to provide a point for the dialysis solution to enter and leave the peritoneal cavity during peritoneal dialysis.^41^ It provides good access to the peritoneal cavity for chronic peritoneal dialysis and treatment of intraperitoneal malignancy. There are various methods for placement like blind insertion, open surgical implantation and peritoneoscopic guided placement. Ultrasound guidance is used for safe insertion. Infection is a frequent complication. Contraindications are peritonitis and sepsis.

### Paracentesis vs PVS placement

There is no difference in survival or quality of life between patients treated with repeated abdominal paracentesis and patients treated with a PVS.^32^ In one study, 20 patients underwent PVS placement and 49 patients were subjected to paracentesis (Table 6). PVS placement thus provides an effective treatment option for patients with refractory malignant ascites in advanced cancer, and yields a higher likelihood of discharge compared with conventional paracentesis.^42^

**Table 6:** Differences between paracentesis and peritoneovenous placement.

	Paracentensis	Peritoneovenous placement
Abdominal girth	No significant decrease	Significant decrease
Hematocrit	No significant decrease	Significant decrease
Blood urea nitrogen, creatinine	No significant change	Tended to decrease
Median no. of procedures	Two	One (*P*<.0001)
Postoperative performance score	No significant improvement	Significant improvement (*P*=.0026)
Median survival	18 days	42 days (*P*=.003)
Discharge rates	Lower	Higher (*P*=.0076)
Severe complications	Seven patients	One patient

### Image-guided biopsies

When used in the context of multidisciplinary team discussion, image-guided biopsy using ultrasound (US) or computed tomography (CT) guidance is of value in planning the management of women with suspected ovarian cancer and peritoneal carcinomatosis (PC) of uncertain etiology.^42^ It is essential in women believed to have ovarian cancer, but with poor performance status or with advanced disease believed beyond the scope of primary cytoreductive surgery for which staging surgical pathology will not be obtained. It provides a site-specific primary tumor diagnosis in 93% of cases and should replace diagnostic laparoscopy or laparotomy for this purpose.^43^ The technique is simple, safe and effective and can be combined with palliative drainage of ascites at the same procedure.^43^

### Recommendations

Although abdominal paracentesis, diuretics and peritoneovenous shunting are commonly used procedures in management of malignant ascites, the evidence for these treatment options is weak. There are no randomized controlled trials evaluating the efficacy and safety of these procedures in malignant ascites. Available data show a good but temporary effect of abdominal paracentesis on symptom relief in patients with malignant ascites. There is no consensus on fluid withdrawal speed and concurrent intravenous hydration is not sufficiently studied. Data show that peritoneovenous shunts can control malignant ascites, but have to be balanced by the potential risks of this procedure. The use of diuretics should be considered in all patients, but has to be evaluated individually. A recommendation for further research is a randomized controlled trial comparing the use of diuretics with paracentesis in the management of malignant ascites.^34^ Guidelines in the management of symptomatic malignant ascites in advanced cancer. Paracentesis is indicated for those patients who have symptoms of increasing intra-abdominal pressure. Available data show good, although temporary relief of symptoms in most patients. Symptoms like discomfort, dyspnea, nausea and vomiting seem to be significantly relieved by drainage of up to 5 L of fluid. When removing up to 5 L of fluid, intravenous fluids seem to be not routinely required (grade of recommendation: D) (Grading of the evidence and the recommendations in the guideline are based on the revised grading system by the Scottish Intercollegiate Guidelines Network (SIGN). Grades of recommendations are from grade A to D. Grade A: At least one meta-analysis, systematic review, or RCT rated as 1++ and directly applicable to the target population. Grade B: A body of evidence including studies rated as 2++ directly applicable to the target population and demonstrating overall consistency of results. Grade C: A body of evidence including studies rated as 2+ directly applicable to the target population and demonstrating overall consistency of results. Grade D: Evidence level 3 or 4 or Extrapolated evidence from studies rated as 2+).^44^ If patient is hypotensive or dehydrated or known to have severe renal impairment and paracentesis is still indicated, intravenous hydration should be considered. Infusion therapy is not sufficiently studied. The only investigated therapy in malignant ascites is infusion of dextrose 5%. There is no evidence of concurrent albumin infusions in patients with malignant ascites (grade of recommendation: D). To avoid repeated paracenteses, peritoneovenous shunting may be considered. Major complications (pulmonary edema, pulmonary emboli, clinically relevant disseminated intravascular coagulation and infection) have to be expected in about 6% of patients (grade of recommendation: D). There are no randomized controlled trials assessing the efficacy of diuretic therapy in malignant ascites. The available data are controversial and there are no clear predictors to identify which patients would benefit from diuretics. The use of diuretics therefore should be considered in all patients, but has to be evaluated individually. Patients with malignant ascites due to massive hepatic metastasis seem to respond more likely to diuretics than patients with malignant ascites caused by peritoneal carcinomatosis or chylous ascites (grade of recommendation: D).^32^

## Newer pharmacological approaches in the management of malignant ascites

### Intraperitoneal Immunotherapy

#### Interferon alpha

One study reported that there was resolution of malignant ascites in 3 of 10 patients treated with intraperitoneal interferon alpha-2b.^45^ Another study showed a 36% complete response and 9% partial response with intraperitoneal administration of interferons in patients with ovarian cancer.^46^ The only significant frequently occurring side effect was pyrexia. No significant myelosupression was observed.^46^ Parenteral interferon has also shown to give positive results in one small study in which intramuscular interferon was given to 5 patients with advanced ovarian carcinoma.^47^ Ascitic fluid production stopped in 2 patients, while disease remained stable in more than 1 year in 2 others and was improved in the fifth patient.

#### Tumor necrosis factor-alpha

Tumor necrosis factor (TNF)-alpha has been shown to be effective in the palliative treatment of malignant ascites.^48^ One study showed a good response to TNF-alpha therapy in patients with malignant ascities. Of 22 patients, 16 had complete and 6 had partial resolution of their ascites. The response rate was highest in patients with ovarian cancer in which the tumor load was distributed in fine nodules all over the peritoneal cavity rather than as palpable bulky masses characteristic of non-ovarian tumors. Some reversible adverse affects such as fever, chills, nausea, vomiting, and fatigue were reported, but these were generally well tolerated.

#### OK-432

A large Japanese study showed favorable results in the use of intraperitoneal injections of streptococcal antigen OK-432 in patients with malignant ascites.^49^ Of 200 patients with malignant ascites of gastrointestinal malignancies, 150 were randomly selected to be administered 6 intraperitoneal injections of OK-432 at intervals of 1 week, while the remaining 50 patients served as control subjects. Of the 150 treated patients, 76 had a complete response (inability to drain any fluid) and 8 had a partial response (decrease in abdominal girth of >10 cm), giving a total response of 56%. Furthermore, the OK-432 group had a better survival time (10.2 months) compared with the control group (3.1 months). Although each of the above studies had limitations, they suggest that intraperitoneal immunotherapy may have a role in the future management of malignant ascites.

#### Anti-VEGF therapy

Decreasing permeability of vessels by inhibiting the tyrosinase kinase activity of vascular endothelial growth factor (VEGF) receptors has recently been shown to inhibit the formation of malignant ascites in animal models.^50^ In that study, the tyrosine kinase inhibitor PTK 787 was evaluated in 2 ovarian cancer cell types. Hey-A8 cells express low levels of VEGF and grow as solid tumor foci on the surface of peritoneal organs, whereas SKOV3 i.p.1 cells express high levels of VEGF and grow as peritoneal tumors and ascites. Treatment of nude mice by means of daily oral administration of 50 mg/kg of PTK 787 was not effective against Hey A-8 tumors, but significantly inhibited the growth of SKOV3 i.p.1 cells and the formation of malignant ascites. Furthermore, survival was increased in SKOV3 mice.^51^ These findings suggest that blockage of the VEGF/vascular permeability factor receptor may be a useful strategy for inhibiting the formation for malignant ascites. This conclusion is further supported by a second study in mice using VEGF-neutralizing antibodies.^51^ Human studies will now be required to test this potentially promising approach toward the management of malignant ascites.

#### Metalloproteinase inhibitors

Encouraging results have also been reported with the intraperitoneal instillation of the metalloproteinase Batimastat.^52^ Twenty three patients with malignant ascites had Batimastat instilled into the peritoneal cavity after paracentesis. No reaccumulation of ascites occurred after that single dose in 5 of the 23 patients, and these 5 survived for up to 112 days. Seven other patients died without reaccumulation. Nausea and vomiting were noted in the first 24 hours after batimastat treatment, but overall tolerance was good, and no significant acute peritoneal reactions were reported. Opposite results, however, were obtained in an animal study in which treatment caused dramatic tumor cell consolidation and less dispersed ascites cells compared with controls, but did not reduce ascites.^53^ It is thus suggested that larger, controlled trials will be necessary before metallopreoteinase inhibitors can be recommended for routine clinical use.

### Radioimmunotherapy

Recently, monoclonal antibody therapy has been used in treating malignant ascites with some success. Five patients with colon or ovarian cancer or mesothelioma were treated with intraperitoneal monoclonal antibody radiolabelled with 131I.^53^ Three of the four assessable patients had resolution of ascites for a mean of 4 months, such that no further paracentesis or diuretic therapy was required. A phase I/II study using a novel anti-mucin monoclonal antibody 2G3 labelled with 131I12 was conducted on 11 patients with chemo-resistant ascites, secondary to ovarian or breast cancer.^54^ The radioimmunotherapy was given by intraperitoneal injection and in three of the four patients who received the highest doses temporary palliation of their ascites lasting 6 weeks to death at 4 months was reported.

### Octreotide

Octreotide, a somatostatin analogue, has been used in the symptomatic management of bowel obstruction, uncontrolled diarrhea and fistulae. It decreases the secretion of fluid by the intestinal mucosa, and increases water and electrolyte reabsorption.^55^ In a study of this agent given subcutaneously in doses ranging from 200 to 600 mcg/24 h to three patients with metastatic adenocarcinoma and ascites, Cairns found that two had a reduction in ascites such that further paracentesis was not required.^55^ More evidence is needed for the establishment of its efficacy in malignant ascites.

## Conclusion

Ascities is a common finding in gastrointestinal malignancies. The effective management of ascities is necessary in treating the symptoms of these patients. We most use ascitic fluid drainage as the most common intervention. Other treatment modalities are specific for the type of malignancies present in the body. Radio- and immunotherapy and other anti-tumor therapies have been used, but none are fully successful in the management of ascites in these patients.
